# Effect of immunonutrition on colorectal cancer patients undergoing surgery: a meta-analysis

**DOI:** 10.1007/s00384-017-2958-6

**Published:** 2018-01-15

**Authors:** Jing Xu, Xian Sun, Qianqian Xin, Ying Cheng, Zhen Zhan, Junfeng Zhang, Juan Wu

**Affiliations:** School of Medicine and Life Sciences, Nanjing University of Chinese Medicine, Box 21, 138 Xianlin Road, Nanjing, 210023 China

**Keywords:** Immunonutrition, Colorectal cancer, Meta-analysis, Surgery

## Abstract

**Purpose:**

Immunonutrition has been used to prevent the complications after colorectal elective surgery. This systematic review aimed to analyze and assess the effect of immunonutrition on colorectal cancer patients who received elective surgery.

**Methods:**

Three electronic databases (Medline, Embase, Cochrane) were used to search the latent studies which investigated the effects of enteral immunonutrition (EIN) compared with standard enteral nutrition (EN) or parenteral immunonutrition (PIN) compared with standard parenteral nutrition (PN) on colorectal cancer patients who are undergoing surgery until 21st of April, 2017. Meta-analysis was conducted to calculate odd risk (OR), mean difference (MD), or standard mean difference (SMD) with 95% confidence interval (CI), and heterogeneity was tested by Q test.

**Results:**

Nine publications were included. The meta-analysis results presented that EIN improved the length of hospital stay (pooled MD, 2.53; 95% CI, 1.29–3.41), infectious complications (pooled OR, 0.33; 95% CI, 0.21–0.53) which contains the Surgical Site Infections (pooled OR, 0.25; 95% CI, 0.22–0.58) and Superficial/Deep incisional infections (pooled OR, 0.27; 95% CI, 0.12–0.64); meanwhile, PIN improved the length of hospital stay (pooled MD, 2.66; 95% CI, 0.62–4.76), IL-6 (pooled MD, − 6.09; 95% CI, − 10.11 to − 2.07), CD3 (pooled MD, 7.50; 95% CI, 3.57–11.43), CD4 (pooled MD, 5.47; 95% CI, 2.54–8.40), and CD4/CD8 (pooled MD, 0.50; 95% CI, 0.22–0.78); the level of CD8 was lower (pooled MD, − 4.32; 95% CI, − 7.09 to − 1.55) in PIN.

**Conclusion:**

Immunonutrition could be an effective approach to enhance the immune function of colorectal cancer patients undergoing elective surgery and to improve the clinical and laboratory outcomes.

**Electronic supplementary material:**

The online version of this article (10.1007/s00384-017-2958-6) contains supplementary material, which is available to authorized users.

## Introduction

Colorectal cancer is one of the most commonly diagnosed cancers in the world [1]. Although early colorectal cancer patients could be treated successfully by surgery, major operation itself possibly cause the dysfunction of the host homeostasis, defense mechanisms and inflammatory response, which would increase the rate of postoperative complications and prolong hospital stay [2, 3]. As nutritional status being a key factor to influence the clinical outcomes, nutrition support has been widely used for elective colorectal surgery patients. Recently, many researchers argued that immunonutritional formulas supplemented with biologically active nutrients were more effective than standard nutrition intervention in improving inflammation, promoting the wound healing and shortening the length of hospital stay (LOS) after operation.

The nutrients of immunonutrition formula usually include arginine, omega-3 fatty acid, glutamine and RNA, etc. Omega-3 fatty acid could reduce the platelet-adhesive endothelial interactions and the synthesis of proinflammatory eicosanoids, while it could stimulate the produce of glutathione which can decrease oxidative injury [4–6]. Arginine is the sole substrate for nitric oxide (NO) synthesis, which is a crucial element of innate antimicrobial immunity in the host’s first line of defense [7]. It also plays an important role in maintaining the physiological balance of gastrointestinal tract [8] and regulating the metabolism of many kinds of lymphocyte [9]. Glutamine, as the major fuel source for macrophages, lymphocytes, and enterocytes, could increase the level of gut mucosal glutathione, thereby reduce free radical availability, and decrease inflammation [10, 11]. The protective effect of glutamine on intestinal mucosa might be attributed to the induction of heat shock protein (HSP) synthesis. Enhanced expression of HSPs (in particular HSP70) has been shown to be responsible for glutamine-mediated cellular protection after inflammatory cytokine-induced cellular injury [12–14]. Deficiency of glutamine may lead to impaired immune function and dysfunction of intestinal epithelium [15].

Although immunonutrition has been used in clinics for more than 20 years, the findings have not been uniform in all reports nor conclusive. For example, one literature approved to use immunonutrition to patients undergoing major surgery regardless of their baseline nutritional status [16], while two literatures suggested that immunomodulating diets have no quantifiable efficacy in well-nourished patients [17, 18]. Senkal et al. [19] revealed a significant reduction of complications receiving immunonutrition on day 3, while Lobo et al. [20] revealed that enteral immunonutrition formula had no advantage over traditional EN formula.

To date, meta-analysis has focused on immunonutrition with digestive system cancer and upper gastrointestinal surgery patients [21, 22], but pooled results about immunonutrition on colorectal cancer patients are still lacking. This systematic review and meta-analysis were done to evaluate whether immunonutrition could be beneficial to colorectal cancer patients, and the conclusion will provide a higher level evidence regarding usage of immunonutrition on colorectal cancer patients undergoing surgery.

## Methods

### Search strategy

The meta-analysis was performed in accordance with the PRISMA guidelines. This systematic review was registered with the International Prospective Register of Systematic Review (PROSPERO), and the registration number is CRD42016049748. Potential studies were searched on Medline (via PubMed), Embase (via OVID), and Cochrane Central Register of Controlled Trials (CENTRAL) from inception to April 2017. The terms and keywords were as follows: (“colon/rectal/colorectal neoplasms”) OR (“colon/rectal/colorectal cancer”) OR (“colon/rectal/colorectal adenomas”) AND (“nutritional support”) OR (“nutrition”) OR (“nutritional sciences”) OR (“arginine”) OR (“glutamine”) OR (“omega-3 fatty acid”) OR (“RNA”) AND (“parenteral nutrition”) OR (“enteral nutrition”) OR (“immunonutrition”). Appropriate Medical Subject Heading (MeSH) terms were combined in the search builder. The results were imported into the management software Endnote X7 to extract data and delete duplicate references.

### Study selection

The inclusion criteria for this study were as follows: (i) studies designed as randomized controlled trials (RCTs), (ii) patients with colorectal neoplasms who received surgery, (iii) intervention of trials was EN vs EIN; PN vs PIN, (iv) both EIN and PIN included at least one of the following nutrients: Arginine, Glutamine, Omega-3 fatty acid.

Studies beyond the inclusion criteria or originally published in language other than English or Chinese were excluded.

### Data extraction and quality assessment

The information and data in all eligible studies were extracted: basic characteristics of each study (first author, publication year, country, sample size, age of the participants), study design (elements in immunonutrition formula, nutritional support duration, approach of the nutritional support), and outcomes of interest (clinical outcomes, immune and biochemical indices).

The quality of included investigations was assessed according to the Cochrane Collaboration tool published in the Cochrane Handbook for Systematic Reviews of Interventions (Version 5.1.0). “Risk of bias” consisting chiefly of six domains: random sequence generation, allocation concealment, blinding of participants and personnel, blinding of outcome assessment, incomplete outcome data, selective reporting, and other bias. Each domain was graded as “low risk of bias,” “high risk of bias,” or “risk of bias unclear.”

### Statistical analysis

Statistical analysis was performed by Revman 5.3. For continuous outcome, mean and standard deviation (SD) of each study was extracted for calculating pool effect. One study conducted by Moya [23] described continuous data by mean and standard error (SE); we transferred it into SD through formula: SD = SE × √*N* (*N* = sample size). Mean difference (MD) was used as effect size when the unit and order of magnitude adopted in all studies were consistent; otherwise, standardized mean difference (SMD) was used. Odds ratio (OR) and 95% confidence interval (CI) were adopted as effect size for dichotomous outcome; events number in intervention and control group was extracted for calculating pooled effect. If effective rate was used, we transferred it into events number by sample size. If multiple intervention patterns were implemented in one study, we took them as multiple independent studies. Outcomes of continuous and dichotomous data were measured using fixed-effect model or random-effect model. The heterogeneity among eligible studies was tested by Q test based on chi-square distribution and *I*^2^ value. Fixed-effect model was used if no significant heterogeneity (*I*^2^ < 50%) existed among studies; otherwise, random-effect model was applied. We removed one or two studies to explore the heterogeneity source and recombined the remaining data to assess the sensitivity of the results. *P* value < 0.05 was considered statistically significant.

## Results

**Fig. 1:**
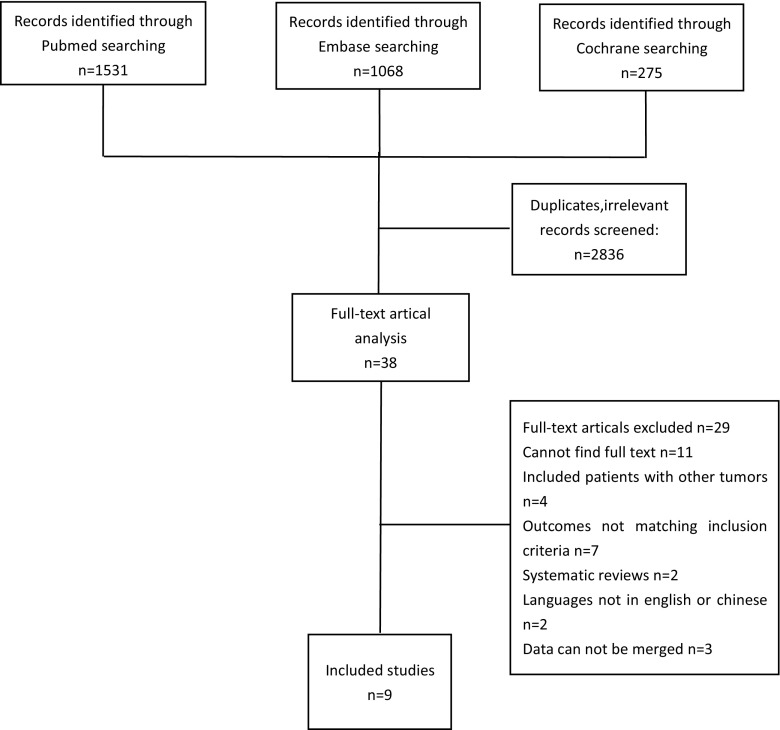
Flow diagram of the literature search and studies selection process

Two thousand eight hundred seventy-four studies were screened out through the initial search: 1531 from Medline database, 1068 from Embase database, and 275 from CENTRAL. After removal of duplicates and irrelevant articles, 38 full-text articles remained of which 26 studies were excluded for not meeting the inclusion criteria. Three of the remaining 12 studies were excluded with data unable to synthesize. Eventually, nine studies [23–31] (six of the EN vs EIN and three of the PN vs PIN) were included for this meta-analysis (Fig. 1).

### Study characteristics

Characteristics of nine studies were outlined in Table 1. In summary, perioperative (*n* = 4) [23–25, 28], preoperative (*n* = 1) [26], and postoperative (*n* = 4) [27, 29–31] interventions were included in this meta-analysis. Six studies taken EN vs EIN [23–28] and three taken PN vs PIN [29–31]. Laboratory indicators including biochemical indices and immune indices (e.g., T cell subsets, cytokines, and immunoglobulin) were measured both before and after the surgery in EN/EIN; in PN/PIN, they were measured 1 day and 1 week after the surgery, respectively; the clinical outcomes including LOS, readmission, and complications were measured within 30 days after surgery.

**Table 1:** Characteristics of the included studies

Author [Ref]	Year	Country	Duration of supplementation	Lost to follow up (Con / IN)	Nutrients	Approaches	Outcomes			
							before sugery	1 day after surgery	1 week after surgery	0~30 days after surgery
Pedro Moya [24]	2016	Spain	7 days prior to surgery and 5 days after surgery	6/7	Arginine,RNA,n3FA	EN Oral	Albumin,prealbumin,transferrin		Albumin,prealbumin,transferrin	Readmission,anastomotic leak,ileus,Infectious complications,Urinary tract infections,Superficial/Deep incisional infetious,Organ/Space infectious,SSI,Respiratory infections
Pedro Moya [23]	2016	Spain	7 days prior to surgery and 5 days after surgery	0/0	Arginine,RNA,n3FA	EN Oral				Readmission,wound infection,Anastomotic leak,ileus,Infectious complications,Respiratory infections
L.S.Sorensen [25]	2013	Denmark	2 days prior to surgery and 7 days after surgery	0/0	n3FA	EN Oral				Readmission,wound infection,Anastomotic leak,ileus,Urinary tract infections,Respiratory infections
Hisanaga Horie [26]	2006	Japan	From 6 to 2 days before surgery	NG	Arginine,RNA,n3FA	EN Oral			albumin	Anastomotic leak,ileus,Urinary tract infections,Superficial/Deep incisional,Organ/Space,SSI,Respiratory infections
Rong Chen [27]	2005	China	From 1 to 7 days after surgery	NG	Glutamine,Arginine,n3FA	EN Nasointestinal tube	albumin,prealbumin,Transferrin,CD4,CD8		albumin,prealbumin,Transferrin	
Marco Braga^1^ [28]	2002	Italy	5 days prior to surgery	0/0		EN Oral				LOS,Anastomotic leak,Infectious complications
Marco Braga^2^ [28]	2002	Italy	5 days prior to surgery and 4 days after surgery	0/0		EN Oral,jejunal infusion				LOS,Anastomotic leak,Infectious complications
Mingwei Zhu [29]	2012	China	From 1 to 8 days after surgery	0/0	n3FA	PN/peripherally-inserted central catheter		CD4,CD8,CD4/CD8,IL-6,TNFα	CD4,CD8,CD4/CD8,IL-6,TNFα	
Bin Liang [30]	2008	China	From 1 to 7 days after surgery	0/1	n3FA	PN/central venous catheter/Peripheral catheter		CD4,CD8,CD3,CD4/CD8,IL-6,TNFα	CD4,CD8,CD3,CD4/CD8,IL-6,TNFα	
Jingxiang Song [31]	2004	China	From 2 to 7 days after surgery	NG	Glutmine	PN/Peripheral venous catheter		CD4,CD8,CD3,CD4/CD8	CD4,CD8,CD3,CD4/CD8	

*Ref* reference, *Con* control, *IN* immunonutrition, *Marco Braga*^*1*^ preoperative, *Marco Braga*^*2*^ peri*-*operative, *NG* not given, *n3FA* omega-3 fatty acids

Nine studies provided a total of 1004 participants diagnosed with colorectal cancer, including 866 participants received the EN/EIN nutrition support and 138 received the PN/PIN nutrition support. In the EN/EIN group, the ratio of control group and intervention group is 433:433; the ratio of control group and intervention group in PN/PIN group is 69:69. More characteristics of participants such as age, gender, weight, BMI, and the situation of metastasis were listed in Table 2.

**Table 2:** Patients characteristics of the included studies

	Moya 2016 [24]	Moya 2016 [23]	Sorensen 2013 [25]	Horie 2006 [26]	Chen 2005 [27]	Braga 2002^1^ [28]	Braga 2002^2^ [28]	Zhu 2012 [29]	Liang 2008 [30]	Song 2004 [31]
Variables (control/intervention)										
Sample size	128/129	61/61	74/74	34/33	36/36	50/50	50/50	28/29	21/21	20/20
Age*	68/70	68/69	71/69	63/69	58.1/57.9	61.8/63.0	61.8/60.5	70.8/69.8	59.19/55.80	56
Sex (male/female)										
Control	69/53	27/34	36/38	18/16	18/18	31/19	31/19	11/17	15/6	26/14
Intervention	62/60	30/31	44/30	25/8	20/16	30/20	8/22	16/13	10/10	
BMI	26.64/27.05	NG	26/26	22.8/22.8	NG	NG	NG	23.2/22.9	23.92/23.38	NG
Metastasis	13/15	NG	5/5	NG	NG	NG	NG	0/0	0/0	NG
Weight	NG	NG	76/77	58/59	59/62	NG	NG	NG	65.4/63.50	NG

*Braga 2002*^*1*^ preoperative, *Braga 2002*^*2*^ perioperative, *NG* not given

*Age is presented by either mean or median

### Quality assessment

Most studies had a clear description of their random sequence generation. Three studies used a computer random number generator [28–30], one used an envelope [25], two used the web-based randomization [23, 24], and three studies did not give the sufficient information [26, 27, 31]. Among them, three studies appropriately performed the allocation concealment [23–25]. Blinding of participants and personnel was conducted in four studies [25, 28–30]. Blinding of participants and personnel was conducted in five studies [25–28, 30, 31]. The remaining studies had no sufficient information about blinding. Two studies reported the drop-out before conducting the immunonutrition [24, 30]; therefore, corresponding domain was graded as “low risk.” All nine included studies showed the pre-specified outcomes in the pre-specified way. The assessment of risk of bias outcome of each study is summarized in Tables 3 and 4.

**Table 3:** Risk of bias summary

	Braga 2002	Chen 2005	Horie 2006	Liang 2008	Moya 2016	Moya 2016	Song 2004	Sorensen 2013	Zhu 2012
Random sequence generation (selection bias)	low risk	unknown risk	unknown risk	low risk	low risk	low risk	unknown risk	low risk	low risk
Allocation concealment (selection bias)	unknown risk	unknown risk	unknown risk	high risk	low risk	low risk	unknown risk	low risk	unknown risk
Blinding of participants and personnel (performance bias)	low risk	unknown risk	unknown risk	low risk	high risk	high risk	unknown risk	low risk	low risk
Blinding of outcome assessment (detection bias)	low risk	low risk	unknown risk	low risk	high risk	high risk	low risk	low risk	unknown risk
Incomplete outcome data (attrition bias)	low risk	low risk	low risk	low risk	low risk	low risk	low risk	low risk	low risk
Selective reporting (reporting bias)	low risk	low risk	low risk	low risk	low risk	low risk	low risk	low risk	unknown risk
Other bias	low risk	high risk	low risk	low risk	low risk	low risk	low risk	low risk	low risk

**Table 4:** The percentage of each bias for all the included studies

	Low risk (%)	High risk (%)	Unknown risk (%)
Random sequence generation (selection bias)	66.67	0	33.33
Allocation concealment (selection bias)	33.33	11.11	55.56
Blinding of participants and personnel (performance bias)	44.44	22.22	22.22
Blinding of outcome assessment (detection bias)	55.55	22.22	22.22
Incomplete outcome data (attrition bias)	100	0	0
Selective reporting (reporting bias)	88.89	0	11.11
Other bias	88.89	11.11	0

### Quantitative data synthesis

#### Effect of enteral immunonutrition on clinical outcome indicators after surgery

**Fig. 2:**
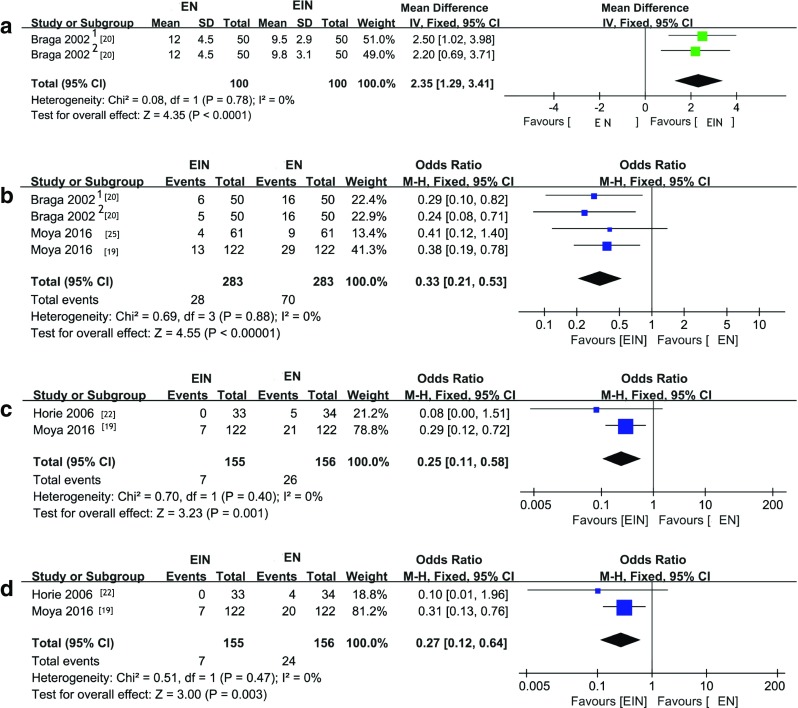
Forest plot comparison between EN and EIN for clinical index. **a** LOS. **b** Infectious complications. **c** SSI. **d** Superficial/deep incisional infections, *Braga 2002*^*1*^ preoperative group, *Braga 2002*^*2*^ perioperative group

The meta-analysis showed that EIN group had a shorter LOS than EN group. Fixed-effect pooled MD was 2.35 (95% CI, 1.29–3.41) with null heterogeneity (*I*^2^ = 0%) (Fig. 2a). Infectious complications were reduced in EIN group for the fixed-effect pooled OR 0.33(95% CI, 0.21–0.53); no heterogeneity was detected (*I*^2^ = 0%) (Fig. 2b). Surgical Site Infections (SSI) and Superficial/Deep incisional infections were reduced in EIN group; the fixed-effect pooled OR was 0.25 (95% CI, 0.11–0.58) in SSI and 0.27(95% CI, 0.12–0.64) in Superficial/Deep incisional infections; no heterogeneity was detected (*I*^2^ = 0%) (Fig. 2c, d). Other outcomes, such as anastomotic leak, ileus, organ/space infections, urinary tract infections, respiratory infections, and readmission, were not significantly different between two groups (Supplementary Table 1).

#### Effect of enteral immunonutrition on laboratory index

No pooled results of laboratory index were found significantly different between EN and EIN in this meta-analysis (Supplementary Table 2).

#### Effect of parenteral immunonutrition on clinical outcome indicators after surgery

**Fig. 3:**
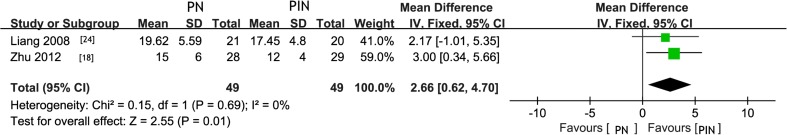
Forest plot comparison between PN and PIN for LOS

PIN intervention could shorten the LOS compared to PN. Pooled MD was 2.66 (95% CI, 0.62–4.76), and the homogeneity was well (*I*^2^ = 0%) (Fig. 3).

#### Effect of parenteral immunonutrition on laboratory index

CD8 and IL-6 were decreased in the PIN group than those in PN group 1 week after the surgery.

**Fig. 4:**
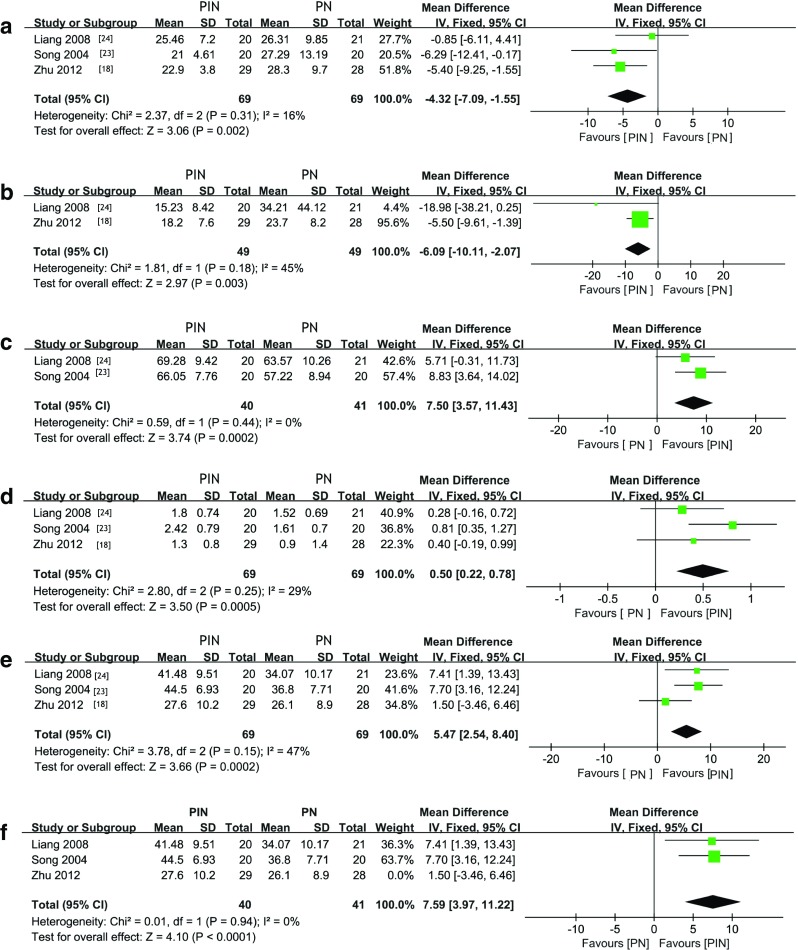
Forest plot comparison between PN and PIN for laboratory index. **a** CD8. **b** IL-6. **c** CD3. **d** CD4/CD8. **e** CD4

Pooled MD for CD8 was − 4.32 (95% CI, − 7.09 to − 1.55) and − 6.09 (95% CI, − 10.11 to − 2.07) for IL-6 (Fig. 4a, b). CD3, CD4/CD8, and CD4 increased in PIN group 1 week after the surgery. Combined MD for CD3 was 7.50 (95% CI, 3.57–11.43), CD4/CD8 was 0.50 (95% CI, 0.22–0.78), and CD4 was 5.47 (95% CI, 2.54–8.40) (Fig. 4c–e). In order to find the source of relative high heterogeneity of CD4, a study conducted by Zhu et al. [29] was removed and *I*^2^ reduced to zero; meanwhile, pooled MD of CD4 level reached 7.59 (95% CI, 3.97–11.22) (Fig. 4f).

## Discussion

In the present meta-analysis, we evaluated the benefits of immunonutrition in patients with colorectal cancer after surgery. Overall, the pooled results supported the usage of EIN in colorectal cancer patients, for the improvement of postoperative complications and reduction of LOS, compared with EN. Meanwhile, PIN strategy also showed to be beneficial for patients’ LOS and cellular immune function parameters. Regarding the proinflammatory factors, the PIN group had a lower serum IL-6 level than that in the PN group, and the increased CD4, CD4/CD8, and CD3 T lymphocytes in the PIN group were reasonably observed, which reflect the enhancing immune function.

Colorectal cancer is the third most commonly diagnosed cancer in males and the second in females, and the mortality rate ranked fourth in males and third in females. Statistics data from IARC showed that the number of new cases of colorectal cancer was 1.4 million and 693,900 cases died in 2012 in the world [1]. Patients of colorectal cancer often suffer from malnutrition, especially those undergoing tumorectomy, and the worsening of nutritional status consequently deteriorated the surgical outcomes. Thus, nutrition intervention has been the focal point of postoperative recovery [32]. Despite the standard nutrition support could reverse nitrogen imbalance and promote patients’ nutritional status, it did little contribution in improving the immune function. Thus, many researchers advocated the usage of immunonutrition in colorectal cancer patients. A growing body of studies suggests that the immunonutrition could reduce the postoperative complications and shorten the LOS in surgical patients, and it also lower toxic effect after the chemoradiotherapy, such as nausea, vomiting, bloating, abdominal pain, diarrhea, or constipation [33]. Immunonutrition formula usually includes arginine, omega-3 fatty acid, glutamine and RNA, etc. Andrade et al. [34] found dietary arginine could preserve the intestinal mucosa and tend to decreased inflammation by histologic analysis. Glutamine supplementation in enteral/parenteral nutrition was able to reduce septic complications, accelerate wound healing, and shorten LOS [35]. However, not all the studies showed the beneficial effects of immunonutrition. For example, Giger-Pabst et al. [36] found that preoperative oral supplementation with an immune-enriched diet for 3 days did not improve postoperative outcome in patients with gastrointestinal cancer, and meanwhile, no positive effects of immunonutrition support were found in ICU patients [37, 38]; furthermore, a study including 1223 critically ill adults showed harmful effects of early administration of immunonutrition [39]. The above results indicated that the effects of immunonutrition would be different when the conditions are different. Patient characteristics of demographic, sample size, control group selection, different administration dose, and duration may be the sources of heterogeneity [40]. In this meta-analysis, the present results confirmed that the immunonutrition support did work for the patients with colorectal cancer.

EN and PN were two different drug delivery routes. In general, EN is prior to PN in clinical practice. PN is used only in patients with the following conditions: not feasible or tolerated for EN, unable to receive and absorb adequate amounts of oral/enteral feeding for at least 7 days due to postoperative complications impairing gastrointestinal function [41]. In this meta-analysis, we also found a different effect between EIN and PIN. EIN mainly improved the clinical outcomes such as SSI and Superficial/Deep incisional infection. However, PIN mainly increase the immune function and reduce inflammation through several laboratory indices, such as raised level of CD4, CD4/CD8, and CD3 T lymphocytes and decreased serum level of IL-6.

Enhanced recovery after surgery (ERAS) protocols are designed to accelerate recovery after surgery [42]. Several studies have demonstrated that ERAS protocols can reduce morbidity and shorten the LOS following colorectal surgery [43–48]. Two of the included studies evaluated benefits of immunonutrition on colorectal cancer patients within an ERAS [23, 24]. The wound infection decreased in both of the two studies. However, reduction of infectious complications occurred only in patients undergoing laparoscopic surgery. Those results indicated the potential effect difference of immunonutrition in patients undergoing laparoscopic and open surgery.

There are different opinions regarding the mechanisms of immunonutrition on immune function and inflammation. Two reports found glutamine could raise secretion of sIgA in the intestine and prevent the translocation of intestinal microbiota [49, 50]. Costa et al. [51] found that supplementation with arginine prevented the increases in intestinal permeability and bacterial transfer caused by exertional hyperthermia and indicated that dietary L-arginine supplementation preserves the integrity of the intestinal epithelium. Those may be the possible explanation of the positive effect of immunonutrition in patients with colorectal cancer in this meta-analysis.

Several strengths and limitations in this meta-analysis should be described. Both EN and PN effects on clinical and laboratory indices were analyzed in this systematic review and meta-analysis, and it could provide the comprehensive evaluation of immunonutrition in colorectal cancer patients after surgery. Even though, there are aspects of this study that can be improved in future. First, dose of nutrients is an important factor for the effect of immunonutrition. Nutrient dose in immunonutrition formula was ranged from 3 to 15.8 g/d [25, 28] in EIN vs EN and from 0.2 to 0.4 g/kg/d in PIN vs PN [30, 31]. In this meta-analysis, we did not explore the dose-response relation, due to the small number of included studies; second, population stratification (e.g., age, gender, and race) was not evaluated due to lacking of enough included studies; third, studies in languages except for English and Chinese were ineligible for inclusion criteria.

## Conclusion

Immunonutrition is beneficial for colorectal cancer patients undergoing surgery. It may decrease the rate of postoperative complications, shorten LOS, and enhance immune function. Immunonutrition could be encouraged in the clinical treatment. More studies with specific timings (preoperative, perioperative, and postperative) are needed for better understanding of immunonutrition in clinical practice. Use of immunonutrition within an ERAS may be more effective. Whether immunonutrition has a long-time effect of patients also needs to be clarified in future.

## Electronic supplementary material

ESM 1 (DOCX 12 kb)
