# Supplementary material for: Effect of immunonutrition on colorectal cancer patients undergoing surgery: a meta-analysis
Source: Int J Colorectal Dis. 2018 Jan 15;33(3):273–83. doi: 10.1007/s00384-017-2958-6 (PMC5816768; doi:10.1007/s00384-017-2958-6)
Supplement: Supplementary file 1 — (DOCX 12 kb) [file 384_2017_2958_MOESM1_ESM.docx]

S1 forest plot comparison between EN and EIN for clinical index. Abbreviation: *a* Anastomotic leak; *b* Ileus; *c* Readmission; *d* Organ/space infections; *e* respiratory infections; *f* urinary tract infections; *g* wound infections; *braga 2002^1^* Preoperative group; *braga 2002^2^* Peri-operative group

S2 forest plot comparison between EN and EIN for laboratory index. Abbreviation: *a 1)* Alubumin level before surgery；*a 2)* Alubumin level after surgery；*b 1)* Prealubumin level before surgery; *b 2)* Prealubumin level after surgery; *c 1)* Transferrin level before surgery; *c 2)* Transferrin level after surgery

S3 forest plot comparison between PN and PIN for laboratory index. Abbreviation: *a* CD3 level one day after surgery; *b* CD4 level one day after surgery; *c* CD4/CD8 level one day after surgery; *d* CD8 level one day after surgery; *e* IL-6 level one day after surgery; *f 1)* TNF-αlevel one day after surgery; *f 2)* TNF-αlevel one week after surgery
